# Cochlin Induced TREK-1 Co-Expression and Annexin A2 Secretion: Role in Trabecular Meshwork Cell Elongation and Motility

**DOI:** 10.1371/journal.pone.0023070

**Published:** 2011-08-23

**Authors:** Manik Goel, Adam E. Sienkiewicz, Renata Picciani, Richard K. Lee, Sanjoy K. Bhattacharya

**Affiliations:** Bascom Palmer Eye Institute, University of Miami, Miami, Florida, United States of America; Alcon Research, Ltd., United States of America

## Abstract

Fluid flow through large interstitial spaces is sensed at the cellular level, and mechanistic responses to flow changes enables expansion or contraction of the cells modulating the surrounding area and brings about changes in fluid flow. In the anterior eye chamber, aqueous humor, a clear fluid, flows through trabecular meshwork (TM), a filter like region. Cochlin, a secreted protein in the extracellular matrix, was identified in the TM of glaucomatous patients but not controls by mass spectrometry. Cochlin undergoes shear induced multimerization and plays a role in mechanosensing of fluid shear. Cytoskeletal changes in response to mechanosensing in the ECM by cochlin will necessitate transduction of mechanosensing. TREK-1, a stretch activated outward rectifying potassium channel protein known to act as mechanotransducer was found to be expressed in TM. Cochlin expression results in co-expression of TREK-1 and filopodia formation. Prolonged cochlin expression results in expression and subsequent secretion of annexin A2, a protein known to play a role in cytoskeletal remodeling. Cochlin interacts with TREK-1 and annexin A2. Cochlin-TREK-1 interaction has functional consequences and results in changes in cell shape and motility. Annexin A2 expression and secretion follows cochlin-TREK-1 syn-expression and correlates with cell elongation. Thus cytoskeleton changes in response to fluid shear sensed by cochlin are further mediated by TREK-1 and annexin A2.

## Introduction

A number of late onset and progressive diseases for example, glaucoma and idiopathic intracranial hypertension are associated with fluid flow abnormalities. Cells dynamically responds to fluid shear, however, such mechanosensing and their responses are yet to be well understood at the molecular level. Altered properties of the cells of a filter like structure termed trabecular meshwork (TM) in the anterior eye chamber are thought to cause fluid shear abnormalities leading to aqueous outflow dysregulation, intraocular pressure (IOP) fluctuations and glaucoma [1], [2]. Physiologically how the cells of the TM region sense and respond to different fluid flow regimes controlling geometry and area of the filter remains to be elucidated. Two distinct components are envisaged for regulation of fluid flow: a mechanosensor residing at the extracellular matrix (ECM) and transmembrane mechanotransducers residing at the cell surface.

Cochlin, a secreted protein identified in the glaucomatous, but not normal TM, by mass spectrometry was found to undergo multimerization in response to fluid shear [3]. Cochlin bears two von Willebrand factor A-like (vWFA) domains that are found in fluid shear responsive ECM proteins [4]. Fluid shear induces cochlin multimerization suggesting cochlin to possess mechanosensing capability [3]. Experiments performed in DBA/2J mice, monkey, and porcine cultured anterior segments [5], [6] and normotensive rabbits [7] are consistent with a key role for cochlin in IOP regulation. The fluid flow changes must be sensed by cells in order to regulate the structure of the TM that allows passage of aqueous humor and to regulate its flow. Fluid shear responsive property of cochlin in consonance with transmembrane shear transducing proteins stretch activated channels (SACs) such as TREK-1 [8], [9] is likely to play a role in mechanotransduction and tissue modeling. Stretch-activated channels have been proposed to be ocular barometers [10]. TREK-1 is a mechanosensitive stretch activated potassium channel [11]. TREK-1 is expressed at mRNA level in the TM [accession number GDS 359; gene expression omnibus (GEO) database]. The TREK-1 channel undergoes mechano-, pH- and voltage-dependent gating and also possesses a domain to interact with membrane phospholipids. TREK-1 activation alters the cytoskeletal network, induces actin cytoskeleton remodeling and is involved in formation of actin- rich membrane protrusions [11], [12]. The transduction of mechanosensing by cochlin with TREK-1 is thus plausible, which may lead to remodeling of TM cell cytoskeleton rendering increased passage for aqueous humor. Cell motility and adhesion require a dynamic remodelling of the membrane-associated actin cytoskeleton in response to extracellular stimuli (such as changing fluid shear). Rho-mediated actin rearrangement of TM cells has implicated in regulation of aqueous outflow [13]. A tyrosine phosphorylation switch in annexin A2 has been shown to be an important event in triggering Rho/ROCK-dependent and actin-mediated changes in cell morphology associated with cell adhesion [14], [15]. We have previously determined interaction of cochlin with annexin A2 using mass spectrometry [16]. Annexins are involved in many membrane remodeling events that involve actin cytoskeleton serving as linkers of membrane-cytoskeleton, organization [17]. Annexins are a family of calcium ion dependent phospholipid-binding proteins. Annexin family is comprised of more than 50 members. Each annexin possesses a short variable N-terminal and a conserved C-terminal core domain [14], [17]. We present evidence that cochlin-TREK-1 and cochlin-annexin A2 interaction is commensurate with changes in TM cell shape and motility which ultimately changes the filter like structure of TM affecting aqueous outflow.

## Materials and Methods

### Ethics statement

The work was conducted adhering to the guidelines of the Institute Review board of the University of Miami. All human samples were handled in keeping with the principles expressed in the Declaration of Helsinki. All experiments with the human samples were conducted at the SKB (ocular proteomic laboratory) lab and the protocol was approved by the Institute Review board of the University of Miami. A written informed consent was obtained from all patients undergoing trabeculectomy for primary open angle glaucoma (POAG) and donating the tissue so obtained for research. Cadaver human eyes were obtained from National Disease Research Interchange, Philadelphia with the approval (exempt under category 4 of NIH guidelines) of the Institute Review board of the University of Miami. Human TM cell culture protocol was approved by the Institute Review board of the University of Miami.

### Tissue procurement

Human eyes from normal and POAG donors, all between 40 and 85 years of age (Table S1), were used in this study, and were obtained from the National Disease Research Interchange. Eyes were enucleated within 12 h of death and stored at −80°C until TM tissue was isolated by dissection. Normal control eyes were from donors with no visual field defects, no evidence of glaucoma, and without central nervous system abnormalities. Fixed human TM tissues used for immunohistochemistry were obtained from the Eye Donor Program of the Foundation Fighting Blindness (Owings Mills, MD). Glaucomatous eyes and tissues were from clinically documented POAG donors. Glaucomatous TM tissues (∼1–2 mm^3^) were obtained by trabeculectomy from POAG patients in the BPEI and Mundorf Eye Center (Charlotte, NC) with institutional review board approval. Human tissue obtained by trabeculectomy consisted predominantly of TM; however, possible contamination with small amounts of surrounding tissue (e.g. sclera) cannot be excluded. TM cells for cell culture were isolated from the rim tissue associated with corneas used for transplantation at the BPEI and were obtained from healthy human eyes within 3 h of death and stored until use in Optisol-GS medium (Chiron Vision, Claremont, CA).

### Western blot analysis

Tissues were dissected out of the enucleated eyes, finely minced, and proteins were extracted using 50 mM Tris-HCl, pH 7.5, 125 mM NaCl and 0.1% genapol (cat# 345794, EMD Biosciences, La Jolla, CA). Cells were pelleted and subjected to extraction using the buffer above. The protein extract was subjected to Western blot analysis. For Western blot the proteins were separated on 4–20% Tris-glycine gel (cat# EC6028BOX, Invitrogen, Carlsbad, CA) and then transferred to polyvinylidene fluoride membranes (PVDF) (cat# 162-0219, BioRad Labratories, Hercules, CA). For cochlin identification, custom antibodies against cochlin peptides (KR LKK TPE KKT GNK DC from cochlin coding region 147–162 designated as hCochlin# 1; ZCZ TYD QRT EFS FTD YST KEN; from cochlin coding region 412–429 designated as hCochin#2; and CZ DDL KDM ASK PKE SH from cochlin coding region 358–371 designated as hCochlin#3, Aves Labs Inc., Tigard, OR) were used [16]. A secondary antibody conjugated to horseradish peroxidase (goat anti-chicken cat# H-1004, Aves Labs Inc.) was added and proteins were detected using enhanced chemiluminescent substrate (cat# 32106, Pierce Thermo Fisher Scientific Inc, Rockford, IL). GAPDH (anti-GAPDH cat# ab22556, Abcam, Cambridge, MA) was used as a loading control.

### Bioinformatic Analyses

Analyses were carried out for known and predicted protein interactions for cochlin that include physical (direct) as well as functional (indirect) associations in different databases (MIPS, DIP, MINT and String) which encompass and track protein-protein interaction or co-expression. Additionally, a short list of proteins encompassing basement membrane, stretch activated channels and endoplasmic reticulum was prepared for further investigation. Potential cochlin-interacting proteins or protein co-expression for cochlin was considered from information derived from genomic context, high throughput experiments, conserved co-expression and previous knowledge through data mining these databases. The shortlist consisted of proteins which appeared in more than one database. They were initially selected and retained for further investigation using Western and ELISA analysis if they were expressed in the trabecular meshwork, ear, kidney and/or have been shown to be linked to benign intracranial hypertension.

### Immunohistochemistry

Human TM sections embedded in paraffin were deparaffinized, hydrated for 20 m with 1× phosphate-Buffered Saline (PBS) (cat# 21-040-CV, Mediatech Inc., Manassas, VA ) and blocked in 1× PBS+0.2% bovine serum albumin (BSA) (Fraction V, cat# 2910, EMD Chemicals, Gibbstown, NJ) for 30 m. The primary antibody was added for cochlin and wolframin (*WFS1*), gasdermin (*GSDMB*), diaphonous related formin 1 (*DRF1*) and alpha-tectorin (*TECTA*) in 1∶200 dilution [Cochlin: hCochlin #3 (cat# 5007/5008) Aves Labs Inc., wolframin: cat# sc-47936, Santa Cruz Biotech, Inc., Santa Cruz,CA. alpha-tectorin: cat#sc-18035, Santa Cruz Biotech., Inc., gasdermin: cat#sc-79952, Santa Cruz Biotech., Inc.]. After incubating overnight at 4°C, primary antibody was washed out with 1× PBS+0.2% BSA 3 times for 10 m per wash., Corresponding secondary antibody was added in 1∶1000 dilution (Cy5:, cat#703177155, Jackson ImmunoResearch Laboratories Inc., West Grove, PA; FITC: cat#IMF-1010, cat# IRF-1010, Aves Labs Inc.) and incubated for 1 h at room temperature. The sections were then mounted on glass micro slides (cat# 48300-0205, VWR International, West Chester, PA) and stained with DAPI Vectashield (cat# H-1200, Vector Laboratories). Thus prepared slides were imaged using Leica DM 6000 B confocal microscope (Leica, Inc.).

### Reciprocal immunoprecipitation (IP)

Reciprocal immunoprecipitation for cochlin (hCochlin#3, Aves Labs Inc.) and annexin (annexin A2: cat# sc-1924, Santa Cruz Biotech., Inc.) was carried out using established protocols [16].

### Demonstration of overexpression of wolframin, alpha-tectorin, diaphanous related formin-1 and gasdermin in human glaucomatous TM

The TM protein extract was prepared from human normal and glaucomatous TM as described. The protein extract was subjected to Western blot analysis. The proteins were separated in 4–20% Tris-glycine gel and then transferred to PVDF membrane. For identification of wolframin (*WFS1*), gasdermin (*GSDMB*), diaphonous related formin-1 (*DRF1*) and alpha-tectorin (*TECTA*), the corresponding primary antibodies were used (wolframin: cat# sc-47936, Santa Cruz Biotech. Inc.; gasdermin: cat#sc-79952, Santa Cruz Biotechnology Inc.; DRF1: cat# HPA004916, Sigma-Aldrich; alpha-tectorin: cat#sc-18035, Santa Cruz Biotech. Inc.) at recommended dilutions. A secondary antibody conjugated to horseradish peroxidase (cat# ab6885, Abcam) was added and proteins were detected using enhanced chemiluminescent substrate (cat# 32106, Pierce Thermo Fisher Scientific Inc.).

### TM cell culture experiments

Primary human TM cells were cultured from cadaveric corneo-scleral sections obtained from the Bascom Palmer Eye Bank (BPEI) and Mundorf Eye Centre (Charlotte, NC). The cells were isolated through a blunt dissection of the area containing and adjacent to the canal of Schlemm, followed by 2 h digestion in 1× PBS (cat# 21-030-CV, Mediatech Inc.) suspension of 20% 0.01 µg/µl collagenase-A (cat# LS004194, Worthington, Lakewood NJ). The blunt dissection and the proteolytic treatment were performed inside a 12 well culture plate (cat# 665-180 Greiner Bio-One, Neuburg, Germany). Culture media containing DMEM 1×(cat# 10-017-CM, Mediatech Inc.), 10% heat inactivated fetal bovine serum (FBS) (cat# 35-016-CV, Mediatech Inc.), 0.5% 1.7 mM L-Glutamine (cat# G6392, Sigma-Aldrich), 1% Antibiotic-Antimycotic solution (cat# 30-004-CL, Mediatech Inc.)] was added after 2 h to terminate digestion. A sterile microscopy slide (cat# 56700-194, VMR) was placed on top of the tissue fragment to ensure bottom contact and immobility inside the media well. The sections were cultured at 37°C, in a 5% CO_2_ cell culture incubator. Cultures were washed with 1× PBS 7 days later to remove tissue debris; media change occurred every 3–4 days. Thus obtained cells were trypsin treated (cat# 25-050-Cl, Mediatech Inc.) and accordingly distributed the day before the transfection Transfection complexes were created using a ratio of 0.4 µg/µl of respective vector DNA to the transfection agent (Lipofectamine 2000, cat# 11668-019, Invitrogen), prepared according to the manufacturer's instructions. Following the addition of the complexes to the selected culture wells, the transfection reactions were allowed to take place over a 2.5 h span, after which they were terminated through the addition of cell culture media. Cell assays and immunohistochemical analysis were performed at or within 24 h in order to observe and track the effects of vector expression on morphological changes. Time-lapse microscopy was performed using cells grown on glass bottom chamber slides (cat# 154534, Lab-Tek, Thermo Fisher Scientific Inc., Rochester, NY) using the Zeiss AxioVert 200 M microscope (Carl Zeiss Inc) for the duration of 20 h, with 15 minutes snapshot frequency, initiated at 24 h post-transfection. Immunohistochemical analysis was performed on cells grown on glass 18 mm glass cover slides (cat# 48380-046, VWR), fixed with 4% PFA (USB Corp) at time intervals of 0, 24, and 29 h post-transfection. Fixed cells were initially blocked in 1× PBS+0.2% BSA for 1 h and incubated with the respective primary antibodies for cochlin, TREK1, actin, annexin A2, alpha-tectorin, green fluorescence protein (GFP) and diaphonous related formin-1 in the dilution factor of 1∶200 in 1× PBS+0.2% BSA overnight (Cochlin: hCochlin #3, cat# 5007/5008; Aves Labs Inc.; TREK-1: cat# ab83932, Abcam; Actin: secondary probing complexed with phalloidin; annexin A2: cat# sc-1924, Santa Cruz Biotech. Inc.; alpha-tectorin: cat# sc-18035, Santa Cruz Biotech. Inc., GFP: cat# 600-301-215, Rockland Inc., diaphonous related formin-1: cat# HPA004916, Sigma-Aldrich). Subsequently, the coverslips were washed three times in 1× PBS+0.2%BSA for 10 m, and incubated with corresponding secondary antibodies for 1 h diluted 1∶1000 in 1× PBS+0.2% BSA (FITC: cat# IMF-1010, Aves Labs Inc.; Rhodamine/Phalloidin: cat# R415, Invitrogen; A546: cat# A10040, A11056, Invitrogen; A594: cat# A11042, Invitrogen; A647:cat# A21449, A21446, A31573, Invitrogen, Cy5: cat# 711-176-152, Jackson ImmunoResearch Labs. Inc.), mounted on glass slides (cat# 48300-025, VWR), stained with DAPI Vectashield (Vector Labs), and finally, imaged using confocal microscopy (Leica DM6000 B) at magnification of 40×/63× with oil immersion. Media for excreted protein analysis was collected at 24 h post transfection and subjected to ELISA probing for cochlin (hCochlin#3, Aves Labs Inc.) and annexin A2 (Santa Cruz Biotechnology Inc).

HEK-293T [cat# 293T/17 (CRL-11268), ATCC, Manassas, VA] and COS-7 [cat# COS-7 (CRL-1651), ATCC] cells were transfected with plasmid expressing cochlin (GeneCopoeia) or annexin A2 [18]. Twenty-four hours post-transfection, media was collected and subjected to Western blot analysis probing for cochlin and annexin A2 with their corresponding primary antibodies (hCochlin#3, Aves Labs Inc, annexin A2: Abcam). The cells were trypsin treated (Mediatech Inc.) and centrifuged at 1,000 rpm for 5 m. The supernatant was discarded and protein extraction buffer (as described for TM protein extraction) was added. The cells were centrifuged again at 10,000 rpm for 10 minutes. The supernatant obtained was then preserved and used for Western blot analysis.

### Methylcellulose gel cochlin release experiment

For a direct, aseptic and localized delivery of gel suspension, a hole was bored through the side of a 35 mm glass bottom culture dish (cat# P35G-1.0-20-C Mat-Tek Corp., Ashland MA), through which a borosilicate capillary (cat# TW100-6, WPI Inc, Sarasota FL) was inserted. The capillary was secured at an angle from the outside using moldable adhesive (cat# 08-0102, Crayola, Shelton, CT) with its outlet fixed to one particular area at the bottom of the culture plate. Prior to plating the cells, the above described setup was UV sterilized and washed several times with 1× PBS to ensure sterile conditions.

TM cells were introduced and allowed to be 50% confluent. Subsequently, the setup was placed inside microscope culture chambers (Zeiss Axiovision 200 M, Carl Zeiss Inc.), maintaining continuous temperature at 37°C and CO_2_ concentration at 5%. A suspension containing 1% methylcellulose suspended in H_2_O (cat# 274445, Sigma-Aldrich) and 10% by volume purified cochlin was prepared and drawn into a 1 ml syringe (cat# 13675-09, Henke-Sass-Wolf, Tuttlingen, Germany) . This suspension was then injected into the external end of the borosilicate capillary and allowed to reach the opposite end. Time-lapse microscopy was initiated with specific aim to focus at the immediate area around the output capillary end and the surrounding cells. The time-lapse was initiated immediately following the introduction of the methylcellulose-cochlin time-released suspension and continued for 20 h (images were captured every 15 minutes).

### Voltage sensitive dye experiment

Human TM cells were cultured as described above in 35 mm glass bottom dishes (Mat-Tek Corp.). At 90% confluence, the cells were transfected with the vector bearing the gene of interest. Thirty six hours post-transfection the cell culture media was replaced with 1× PBS at 37°C (cat#14040, Invitrogen) and the culture was imaged in real time using Leica DMI 16000 CS Inverted Scanning confocal microscope (Leica Inc.) at a fixed 493–596 nm spectral emission-excitation wavelength field, specific to the dye molecule being studied. Following the initial snapshot of selected number of cells, bis-(1, 3-dibutylbarbituric acid) trimethine oxonol [DiBAC_4_(3) or DiBAC] (cat# B-438, Invitrogen) was added to the 1× PBS bathing the cells to a final concentration of 1 µM in the solution. Secondary live snapshot was taken 10 minutes following the addition of DiBAC. The fluorescence intensity was quantified for 100 randomly selected cells before and after DiBAC addition using Leica LAS software (v. 2.3.0, build 5131, Leica Inc.)

### Cochlin and annexin A2 secretion pathway

TM cells were transfected with *COCH* gene carrying vector (GeneCopoeia Inc.). They were incubated in the presence or absence of caspase-1 inhibitor (10 nM) (cat# 60856, AnaSpec, Fremont, CA) or pan-caspase inhibitor (10 nM) (cat# 60861, AnaSpec, Fremont, CA) or Golgi disruptor, brefeldin (5 µg/ml) (cat# 203729-1MG, EMD Chemicals). The concentrations used did not produce cytotoxicity as assessed by trypan blue exclusion. The media was collected 24 h post transfection and subjected to ELISA analysis probing for cochlin and annexin A2.

### Overlay assay

Human TM was used to prepare protein extract as described. TM cells were cultured and transfected with HA-tagged cochlin. 24 h post-transfection the media was collected. 100 µg of protein extract and media (from transfected cells) were subjected to non-reducing 10% SDS-PAGE (Invitrogen). The proteins were transferred onto a PVDF membrane which was washed five times with IX TBS to remove SDS and then soaked in the media containing the recombinant HA-tagged cochlin. The membrane was washed and subjected to probing with antibody against HA (cat# sc-805, Cruz Biotechnology Inc.), stripped and further re-probed with antibody against cochlin (hCochlin#3 antibody, Aves Labs Inc.).

### Oleylamine experiment

Primary TM cells were cultured as described earlier in a 12 well plate (Greiner Bio-One) on top of glass cover-slide (VMR). The cells were transfected with a plasmid containing the GFP tagged cochlin vector (GeneCopoeia, Inc.) as described in the TM cell culture methods. Oleylamine (cat# O7805, Sigma-Aldrich), a known inhibitor of TREK-1 potassium channel function (but not mechanotransduction function) [12] was added to one set of cochlin transfected cells to achieve a final concentration of 5 µM. Another set of cochlin-transfected cells was used as the control. Cells grown in the presence or absence of oleylamine were fixed with 4% paraformaldehyde (USB Corp) after 24 h. The glass cover-slides thus obtained were used for immunohistochemical analysis using confocal microscopy and the methods as described previously.

## Results and Discussion

**Figure 1:**
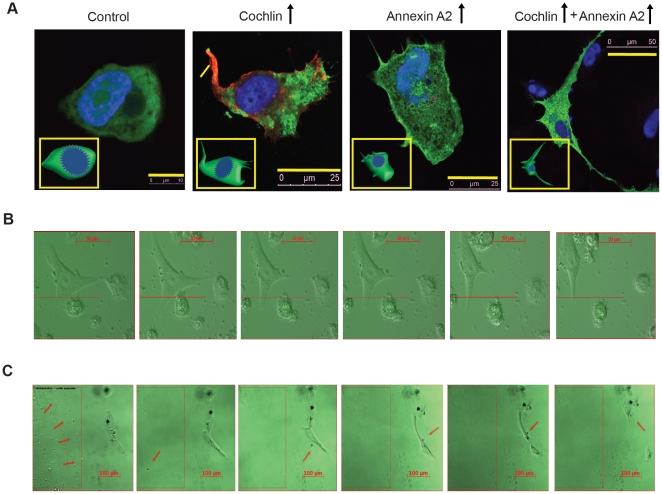
TM cells exposed to high cochlin concentration develop filopodia. **(A)** Representative images of primary TM cells. The TM cells were either left untransfected or were transfected with plasmids expressing cochlin or annexin A2 or both cochlin and annexin A2 as indicated. Nuclei were stained with DAPI (blue). Actin is stained in red and green fluorescence shows GFP expression. Inset shows graphic representation of representative TM cell morphology for the transfection types as described. Scale bar, 10 µm (1st column), 25 µm (2nd and 3rd columns), 50 µm (4th column). **(B)** Morphological changes in a TM cell exposed to cochlin secreted from an adjacent cell transfected with the plasmid expressing cochlin and green fluorescence protein (GFP). Scale bar, 50 µm. **(C)** Morphological changes in a TM cell exposed to a steady sustained release of cochlin from a methylcellulose gel pellet (arrows and semicircle) impregnated with purified recombinant cochlin. Arrows track the course of cochlin vesicles from methylcellulose gel to the TM cells. Scale bar, 100 µm.

Mechanotransduction may induce alterations in cell shape and motility within the TM. Cultured primary human TM cells were used to demonstrate the effects of cochlin, TREK-1 and annexin A2 on cellular morphology. Control untransfected TM cells show straight smooth edges. In contrast, cochlin, annexin A2 and their co-transfection result in sharp, thin and elongated filopodia, stretched cells with multiple hair-like projections and thicker spread out filopodia like structures (Figure 1A). These data suggest that cell shape changes concomitant with cochlin or annexin A2 overexpression. Secreted cochlin from transfected cells modulates the morphology of adjacent untransfected cells (Figure 1B and Movie S1). Cochlin secreting cells show a transparent leading edge (round filopodia), while untransfected cells show filopodia moving away from regions of high cochlin concentration (Movie S1). We next used time-lapse microscopy, exogenous purified recombinant cochlin and TM cells to demonstrate how cochlin concentration gradients promote filopodia formation. Exogenous cochlin laden methylcellulose gel pellets were placed adjacent to TM cells (Figure 1C and Movie S2). TM cells developed elongated filopodia in response to released exogenous cochlin and retracted away from the cochlin gradient (Figure 1C and Movie S2), similar to what was observed for TM cells adjacent to cochlin transfected cells (Figure 1B and Movie S1).

**Figure 2:**
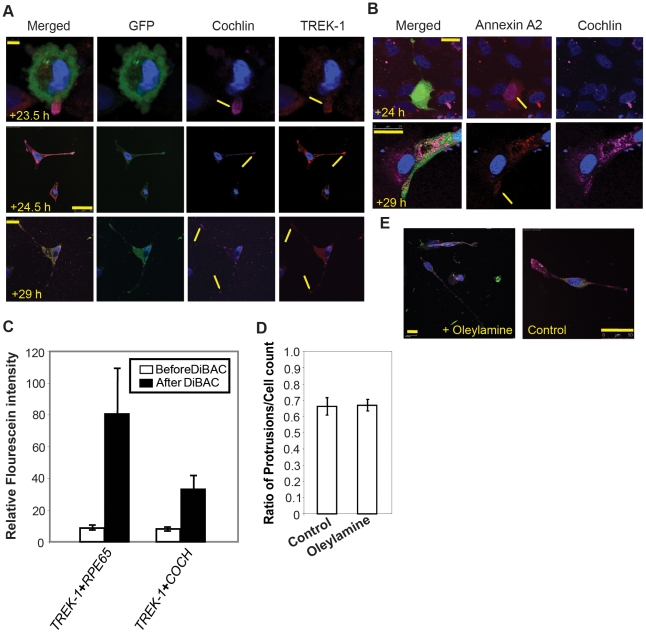
Overexpression of cochlin causes morphological changes in TM cells and cochlin and TREK-1 interaction has functional implications. (A) Immunocytochemical analysis of cells expressing cochlin and green fluorescence protein (GFP). The cells were imaged at 23.5, 24.5 and 29 hours post-transfection as indicated and probed for TREK-1 (red), GFP (green) and cochlin (pink). Arrows show localization of cochlin and TREK-1 in the filopodia. Scale bar, 5 µm (top panel), 50 µm (middle panel), 25 µm (bottom panel). (B) Immunocytochemical analysis of cells expressing cochlin and GFP. The cells were imaged at 24 and 29 hours post-transfection as indicated and probed for annexin A2 (red) and cochlin (pink). Scale bar, 25 µm. (C) Comparison of relative fluorescein intensity in the TM cells transfected with *TREK-1*+ *RPE65* and *TREK-1*+*COCH* before and after the addition of DiBAC as indicated. Results from five different experiments are shown (mean ± SD). (D) Comparison of filopodia induction in the cells cultured in the presence or absence of oleylamine (5 µM). Results from three different experiments are shown (mean ± SD). (E) Representative images of cells expressing cochlin and GFP when grown in the presence or absence of oleylamine (5 µM) as indicated. Scale bar, 50 µm. In all sections nuclei were stained with DAPI.

TM cells demonstrated co-localization of cochlin and TREK-1 at the initial stages of cochlin expression at approximately 23.5 hours post-transfection at the leading edges of the protrusions (Figure 2A). At 24.5 and 29 hours post-transfection, increased expression of TREK-1 was observed at the leading edges of filopodia-like growths. TREK-1 and cochlin expression occurred extensively throughout the cell with increased expression at the leading edges at later stages of cell growth.

Cochlin transfected cells also show localization of cochlin and annexin A2 (Figure 2B and Figure S1A). At early stages of cochlin expression (∼24 hours), annexin A2 localized close to the nucleus; the protein expression shifted toward filopodia at later (29 hours) stages (Figure 2B). These results suggest that cochlin and TREK-1 follow similar expression kinetics with respect to initial filopodia formation. Annexin A2 follows the course of cochlin expression and likely plays a role in filopodia extension and enlargement. The co-expression of cochlin and TREK-1 is consistent with their role in mechanosensing and mechanotransduction.

We next probed functional interaction between cochlin and TREK-1 using the voltage sensitive dye, bis-(1, 3-dibutylbarbituric acid) trimethine oxonol (DiBAC). Activation of TREK-1, an inward rectifying K^+^ channel [19] prevents inward DiBAC transport resulting in lower fluorescence. TM cells transfected with TREK-1+cochlin showed lower fluorescence intensity compared to control TREK-1+ Retinoid isomerohydrolase (*RPE65*) transfected cells incubated in presence of DiBAC (Figure 2C and Figure S2A). Retinoid isomerohydrolase, an ocular protein with a molecular size similar to cochlin was chosen as a control. TM cells transfected with cochlin and cultured in the presence or absence of 5 µm oleylamine, a cationic lipid inhibitor of TREK-1 channel activity [12], showed an unchanged ratio of cells developing filopodia compared to the total number of cells (Figure 2D, E and Figure S2B) suggesting TREK-1 mediated morphological changes in TM cells (Figure 2A) are independent of the channel activity. These findings are consistent with a prior report [12] in other cell types.

Overall, changes in cell shape and motility should create open spaces for fluid outflow. We reasoned that such expansion of the TM slit-like filter should be reflected in cochlin overexpressing TM cells on a matrix. The contribution of cochlin and TREK-1 to space expansion for fluid flow probed using collagen gel assays [20] suggest gel expansion requires the presence of both TREK-1 and cochlin (data not shown).

**Figure 3:**
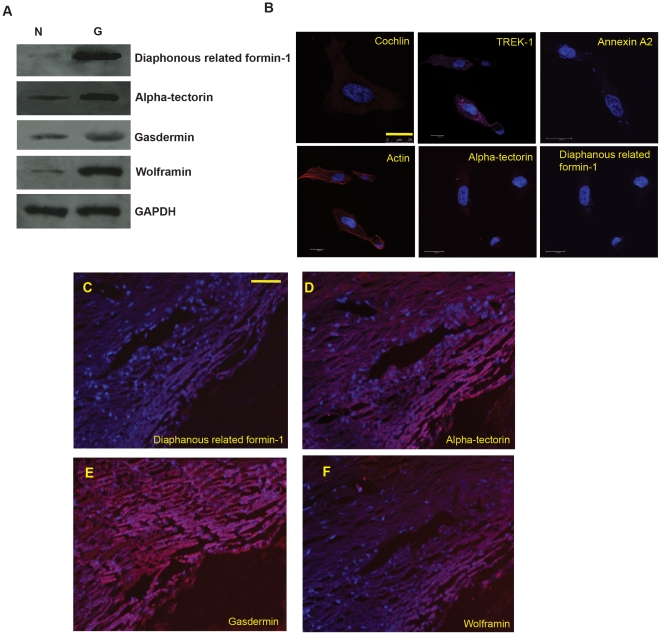
Concomitant elevated levels of select basement membrane and ER proteins with cochlin overexpression. **(A)** Western analysis of normal (N) and glaucomatous (G) human TM protein extract were probed with anti-diaphanous-related formin-1, anti-alpha-tectorin, anti-gasdermin, anti-wolframin and anti-glyceraldehyde 3-phosphate dehydrogenase (GAPDH) antibodies as indicated. Glyceraldehyde 3-phosphate dehydrogenase (GAPDH) is shown as a loading control. Diaphanous-related formin-1 , alpha-tectorin, gasdermin-B and wolframin are expressed in human TM. **(B)** Immunocytochemical analysis of primary TM cells showing the level of expression of cochlin, TREK-1, annexin A2, actin, alpha-tectorin and diaphanous-related formin-1. Scale bar, 25 µm **(C–F)** Immunohistochemical analysis of human TM sections showing the presence of diaphanous-related formin-1, alpha-tectorin, gasdermin-B and wolframin as indicated. Sections were also stained with DAPI to outline the anatomy. Scale bar, 50 µm.

Mechanotransduction in osteocytes and other systems [21], [22], suggests dynamic interplay between secreted and basement membrane proteins for cellular remodeling. Significant modulation in cochlin secretion is likely to be associated with changes in expression of select basement membrane and endoplasmic reticulum proteins. Extensive bioinformatic analyses for known and predicted protein interactions for cochlin in various databases enabled us to short-list select proteins for further investigation, namely: diaphanous related formin-1 (*DRF1*), alpha-tectorin (*TECTA*), gasdermin (*GSDMB*) and wolframin (*WFS1*), which were found to be elevated in human glaucomatous TM (Figure 3A). Diaphanous related formin-1 is usually present in the tip of the membrane ruffles in motile cells, nucleates actins and acts in a Rho-dependent manner. It is required for the assembly of F-actin structures, such as actin cables and stress fibers [23]. Rho-mediated actin rearrangement has been noted in TM cells which have been implicated in regulation of aqueous outflow [13]. Diaphanous related formin-1 is known to regulate actin polymerization in hair cells [23]. Gasdermin, a cytosolic protein have been implicated in playing a role in general in cellular protein secretion, hence it may play a role for secretion of cochlin and annexin. The expression of gasdermin is associated with cell motility as well [24]. Alpha-tectorin is one of the major non-collagenous components of the tectorial membrane. The tectorial-membrane is an extracellular matrix of the inner ear. Alpha-tectorin is implicated in transduction of sound signals in the inner ear. All tectorins are likely synthesized as glycosylphosphatidylinositol-linked, membrane-bound precursors and proteolytically released into the extracellular compartment. Gene expression omnibus database search shows presence of alpha-tectorin at mRNA level in the TM (accession number GDS 359).Like annexin A2, alpha-tectorin is anchored to the membrane with a lipid anchor and released into ECM under certain conditions[25]. Wolframin (WFS1) is a transmembrane protein of the endoplasmic reticulum (ER) that participates in the regulation of cellular calcium ion homeostasis. Elevated levels of wolframin can cause ER stress and elevated intracellular calcium. Defects in WFS1 are the cause of Wolfram syndrome (WFS) also known as diabetes insipidus and mellitus with optic atrophy and deafness syndrome [26], [27]. Analyses confirmed expression of diaphanous related formin-1, alpha-tectorin, actin and co-localization of cochlin, annexin A2 and TREK-1 in normal primary TM cells (Figure 3B) and in tissues (Figures 3C–F).

**Figure 4:**
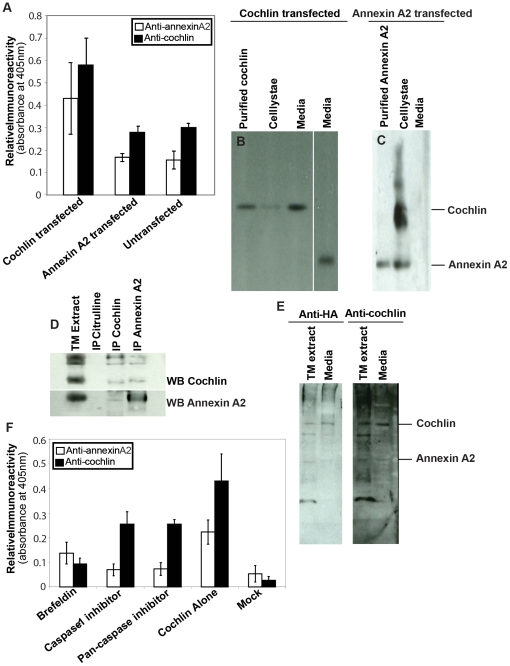
Concomitant expression and secretion of annexin A2 with cochlin overexpression. **(A)** ELISA analysis of the media collected from TM cells transfected with cochlin or annexin A2 or untransfected shows some basal secretion of cochlin and annexin A2 by the TM cells which is greatly augmented by cochlin overexpression but not by annexin A2 overexpression (mean ± SD). **(B–C)** Western blot analysis of the media and cell lysates, collected from HEK-293T cells transfected with cochlin or annexin A2 as indicated. Purified cochlin and annexin A2 are shown as positive controls. **(D)** Reciprocal IP of cochlin and annexin A2 from human TM extract, probed with antibody against either cochlin or annexin A2 as indicated, anti-citrulline IP served as control. IP = Immunoprecipitation; WB = Western blot **(E)** Overlay assay of human TM extract and media from HA-tagged cochlin transfected cells shows both cochlin and annexin A2 when probed with antibodies against HA or subsequently against cochlin as indicated. Cochlin interacts with annexin A2 and augments secretion of the latter by the non-classical pathway. **(F)** ELISA analysis of the media collected from primary TM cells transfected with cochlin shows increased secretion of cochlin and annexin A2 by cochlin transfected cells as compared to mock transfection. Golgi disruptor Brefeldin has a greater effect on cochlin secretion, and caspase-1 inhibitor and pan-caspase inhibitor have greater effect on annexin secretion.

Annexin A2 is a multifunctional protein, associated with morphological changes and elongation in a variety of cell types. If cochlin induces alteration in cell shape and motility, expression of proteins associated with cell elongation, for example, annexin A2 would be anticipated to increase, concomitant with elevated cochlin secretion. Normal TM cells have low secreted cochlin and annexin A2 levels. However, TM cells transfected with *COCH* secrete cochlin, as well as annexin A2. In contrast TM cells transfected with annexin A2 plasmid do not secrete any more annexin A2 than untransfected cells (Figure 4A). HEK-293T and Cos-7 lack basal annexin A2 or cochlin secretion, but when transfected with cochlin constructs, these cells secrete both cochlin and annexin A2 into the media. Recombinant annexin A2 expression in these cells results in elevated annexin A2 in cell lysates but not its secretion (Figures 4B and 4C). Thus, the transfection of cochlin results in secretion of annexin A2, yet annexin A2 overexpression by itself does not lead to its secretion. Previous mass spectrometric analysis indicated the interaction of cochlin with annexin A2 [16]. Cochlin-annexin A2 interactions were demonstrated by reciprocal IP (Figure 4D). In addition to reciprocal IP, we also performed overlay assay which is an independent method to demonstrate protein-protein or protein-lipid interactions [28], [29]. Briefly, the proteins in a tissue or cell lysate, fractionated by electrophoresis and transferred on a membrane are subjected to interaction with target protein which is subsequently immunodetected. Overlay assays further corroborated cochlin-annexin A2 interaction (Figure 4E). These results suggest basement membrane proteins are elevated concomitant with secretion of cochlin in glaucomatous tissue and interaction of cochlin with TREK-1, stretch-activated channel (SAC) proteins and annexin A2 may mediate effects of cochlin.

Using caspase inhibitors as tools for probing non-classical protein secretion [30], we further determined that cochlin is secreted by the classical pathway, in contrast, cochlin induced annexin A2 secretion is likely mediated by the non-classical pathway (Figure 4F). Annexin A2 secretion (but not cochlin) was significantly reduced in HEK-293T cells when incubated with caspase-1 or pan-caspase inhibitor but not with brefeldin (Figure 4F). Brefeldin is an inhibitor of classical protein secretion which inhibits transport of proteins from endoplasmic reticulum [31]. These results suggest that secretion (annexin A2) or levels (diaphanous related formin-1, alpha-tectorin) of select proteins that contribute to cytoskeletal remodeling are modulated by cochlin. Diaphanous related formin-1 expression was found at the leading edges of filopodia, consistent with actin reorganization (Figure S1B) in cochlin transfected TM cells. Similarly diaphanous related formin-1 and alpha-tectorin were also found in the leading edges of TM cells transfected with cochlin (Figures S1C and S1D). These results suggest association of annexin A2 and diaphanous related formin-1 with cell elongation.

Both mono- and multimeric cochlin have been found to interact with SACs such as TREK-1, TASK-1 and choline transporter-like protein 2 (*SLC44A2*). Multimeric cochlin is proteolysis resistant and reside for longer periods in the TM (data not shown). TM cells function in an environment of continuous varying mechanical and fluid shear forces [32]. Our understanding of molecular mechanosensors and their role in transduction of stimuli into cellular biochemical signals that ultimately regulate cellular function [33] is very limited. Reversible multimerization of cochlin caused by shear stress can act as potential mechanosensing mechanisms. The data presented here suggests that mechanical forces are possibly transmitted through cochlin-TREK-1 protein-protein interactions. Our findings link ECM mechanosensing by cochlin with transmembrane mechanotransduction mediated by TREK-1 resulting in modulation of cytoskeletal proteins [34], [35].

The modulation of cell shape and motility and overall space expansion mediated by cochlin should induce overall TM filter changes and result in increased fluid flow. Taken together, these data suggest interaction of cochlin with TREK-1 is involved in formation of filopodia-like protrusions. These processes are likely to contribute to expansion of cells in the gel, consistent with opening spaces between cells and increased transport of sodium fluorescein across cell layer matrix, suggesting a regulatory role for cochlin and TREK-1 in fluid flow across TM tissue. Indeed TM cells overexpressing cochlin but not a control protein Retinoid isomerohydrolase of identical size has been found to contribute to gel expansion and to augment transport of fluorescein across cell layer (data not shown). Our results suggest mechanotransduction by cochlin and TREK-1 of fluid flowing through the ECM can modulate the expression of several cytosolic proteins, for example, diaphanous related formin-1, alpha-tectorin and Profilin I and induce actin cytoskeletal remodeling [36] concomitant with increased outflow facility [37], and reduction of IOP [38], [39].

Our observation of cochlin-TREK-1 co-expression (Figure 2A) is consistent with their initiation of changes in the space between cells within TM filter. The kinetics of annexin A2 appearance is consistent with cytoskeletal remodeling (Figure 2B) and late cellular changes associated with TM remodeling and expansion resulting in enhanced fluid flow across cell layers (data not shown). Maximum resistance to aqueous outflow is localized to the juxtacanalicular tissue of the TM [40]. Studies suggest TM cells dynamically regulate outflow facility by rearranging their cytoskeleton, thus enhancing aqueous outflow [41]. Our results provide insight into the physiology of TM outflow regulation and suggest a role for fluid shear mechanosensing in the aqueous humor outflow process.

## Supporting Information

Figure S1 **Cochlin overexpression alters the morphology of TM cells. (A)** Representative confocal microscopy images of TM cells transfected with a plasmid bearing the GFP tagged *COCH* vector. Images were obtained 24 hours post-transfection and show co-localization of cochlin (pink) and annexin A2 (red) in the filopodia. Scale bar, 25 µm (Top panel), 50 µm (Bottom panel) **(B)** Representative confocal microscopy images of TM cells transfected with a plasmid bearing the GFP tagged cochlin vector. Images were obtained 24 hours post-transfection and show co-localization of cochlin (pink) and actin (red) in the filopodia. Scale bar, 25 µm **(C)** Representative confocal microscopy images of TM cells transfected with a plasmid bearing the GFP tagged cochlin vector. Images were obtained 24 hours post-transfection and show co-localization of cochlin (pink) and diaphanous related formin-1 (red) in the filopodia. Scale bar, 25 µm **(D)** Representative confocal microscopy images of TM cells transfected with a plasmid bearing the GFP tagged cochlin vector. Images were obtained 24 hours post-transfection and show co-localization of cochlin (pink) and alpha-tectorin (red) in the filopodia. Scale bar, 25 µm.
(EPS)

Figure S2 **TREK1 and cochlin interaction alter the membrane potential but the morphogenic effect is independent of channel activity.** **(A)** Comparison of relative fluorescein intensity in the TM cells transfected with *TREK-1*+*RPE65* (top panel) and *TREK-1*+*COCH* (bottom panel) before (left side) and after (right side) the addition of bis-(1, 3-dibutylbarbituric acid) trimethine oxonol (DiBAC). Each colored line represents the data obtained from one cell. The fluorescein intensity is shown on the x-axis and the number of pixels showing that particular intensity is shown on the y-axis. **(B)** Representative confocal microscopy images of TM cells transfected with the plasmid expressing cochlin tagged with GFP grown in the presence (top panel) or absence (bottom panel) of 5 µM oleylamine. Scale bar, 50 µm.
(EPS)

Table S1 **Information of tissue donors.** A categorical and relevant medical details of donors have been provided.
(XLS)

Movie S1 **Exposure to cochlin causes morphological changes in TM cells.** A cochlin transfected TM cell, seen in this video as having a round transparent leading edge and displaying the GFP, releases cochlin into the surrounding media. This high concentration of cochlin in the media causes the cell adjacent to it to develop filopodia in response and move away from the concentration gradient.
(ASF)

Movie S2 **Exogenous cochlin causes TM cells to move away from the concentration gradient.** Cochlin is secreted into the media by the methylcellulose gel loaded with cochlin. As the cochlin containing vesicles reach the TM cells, they bring about a change in the morphological features of the cells. The video here shows how under the influence of cochlin two adjacent TM cells develop filopodia and retract away from each and thus increase the intercellular space.
(ASF)
